# Pannexin 1 Regulates Skeletal Muscle Regeneration by Promoting Bleb-Based Myoblast Migration and Fusion Through a Novel Lipid Based Signaling Mechanism

**DOI:** 10.3389/fcell.2021.736813

**Published:** 2021-10-05

**Authors:** Katia Suarez-Berumen, Henry Collins-Hooper, Anastasia Gromova, Robyn Meech, Alessandra Sacco, Phil R. Dash, Robert Mitchell, Valery I. Shestopalov, Thomas E. Woolley, Sakthivel Vaiyapuri, Ketan Patel, Helen P. Makarenkova

**Affiliations:** ^1^Department of Molecular Medicine, The Scripps Research Institute, La Jolla, CA, United States; ^2^West Anaheim Medical Center, Anaheim, CA, United States; ^3^School of Biological Sciences, University of Reading, Reading, United Kingdom; ^4^Development, Aging and Regeneration Program, Center for Genetic Disorders and Aging Research, Sanford Burnham Prebys Medical Discovery Institute, La Jolla, CA, United States; ^5^Department of Clinical Pharmacology, Flinders University, Adelaide, SA, Australia; ^6^Bascom Palmer Eye Institute, University of Miami School of Medicine, Miami, FL, United States; ^7^Institute for Information Transmission Problems, Russian Academy of Sciences, Moscow, Russia; ^8^Mathematical Institute, University of Oxford, Oxford, United Kingdom; ^9^School of Pharmacy, University of Reading, Reading, United Kingdom

**Keywords:** Panx1, lipid signaling, myoblast migration, pannexins, blebbing, myoblast fusion

## Abstract

Adult skeletal muscle has robust regenerative capabilities due to the presence of a resident stem cell population called satellite cells. Muscle injury leads to these normally quiescent cells becoming molecularly and metabolically activated and embarking on a program of proliferation, migration, differentiation, and fusion culminating in the repair of damaged tissue. These processes are highly coordinated by paracrine signaling events that drive cytoskeletal rearrangement and cell-cell communication. Pannexins are a family of transmembrane channel proteins that mediate paracrine signaling by ATP release. It is known that Pannexin1 (Panx1) is expressed in skeletal muscle, however, the role of Panx1 during skeletal muscle development and regeneration remains poorly understood. Here we show that Panx1 is expressed on the surface of myoblasts and its expression is rapidly increased upon induction of differentiation and that *Panx1^–/–^* mice exhibit impaired muscle regeneration after injury. *Panx1^–/–^* myoblasts activate the myogenic differentiation program normally, but display marked deficits in migration and fusion. Mechanistically, we show that Panx1 activates P2 class purinergic receptors, which in turn mediate a lipid signaling cascade in myoblasts. This signaling induces bleb-driven amoeboid movement that in turn supports myoblast migration and fusion. Finally, we show that Panx1 is involved in the regulation of cell-matrix interaction through the induction of ADAMTS (Disintegrin-like and Metalloprotease domain with Thrombospondin-type 5) proteins that help remodel the extracellular matrix. These studies reveal a novel role for lipid-based signaling pathways activated by Panx1 in the coordination of myoblast activities essential for skeletal muscle regeneration.

## Introduction

Adult mammalian skeletal muscle is composed of multinucleated fibers that cannot divide. Regeneration after an injury is mediated by a resident stem cell population called satellite cells (Lepper et al., 2011; Sambasivan et al., 2011). In undamaged adult muscles, satellite cells are quiescent; however, upon injury, they become activated and divide both asymmetrically and symmetrically. This allows self-renewal of the satellite cell pool, as well as the generation of a transit-amplifying population called myoblasts that become progressively differentiation-competent and eventually withdraw from the cell cycle and differentiate. Muscle regeneration is dependent on the ability of satellite cells to migrate to the point of a lesion, which relies on dynamic cytoskeletal rearrangements and cell-cell communication and is controlled in part by nitric oxide and purinergic signaling pathways (Irintchev et al., 1994; Irizarry et al., 2003; Hutcheson and Kardon, 2009; Makarenkova et al., 2009; Collins-Hooper et al., 2012; Webster et al., 2016). Myoblast migration is characterized by the formation of surface protrusions, called “blebs,” especially on its native substrate, the muscle fiber. Blebs have been implicated in the amoeboid movement of cells that lack mature focal adhesions and stress fibers (Friedl et al., 2001; Friedl and Wolf, 2009; Otto et al., 2011; Khajah et al., 2015). We have previously shown that blebbing is essential to support the rapid migration of myoblasts (Otto et al., 2011). Adhesion of myoblasts to each other and to the extracellular matrix (ECM) and their synthesis of ECM components are also critical for myoblast differentiation (Hantai et al., 1985; Melo et al., 1996; Schwander et al., 2003; Lukjanenko et al., 2016). Myoblasts express a pericellular matrix rich in versican (Ermakova et al., 2011). During differentiation, the pericellular matrix is remodeled by enzymatic cleavage and new matrix synthesis. In particular, the reduction of hyaluronidase-sensitive pericellular coats by the ADAMTS family of versicanases is necessary for myoblast fusion (Hattori et al., 2011; Stupka et al., 2013; Taye et al., 2020).

Muscle fibers and myoblasts express the pannexin family of gap junction-like membrane channel proteins (Panchin et al., 2000; Bruzzone et al., 2003; Baranova et al., 2004; Langlois et al., 2014; Jorquera et al., 2021). The pannexin family consists of three members (Panx1–3; Baranova et al., 2004; Panchin, 2005; D’hondt et al., 2010). Pannexin hemichannels are activated through interaction with purinergic receptors (Iglesias et al., 2008) and by various stimuli such as high intracellular calcium (Locovei et al., 2006), high extracellular potassium (Bunse et al., 2009), membrane stretch via the activation of Piezo-1 channel and submembrane increase in Ca^2+^ signal (Lopez et al., 2021), and membrane depolarization (Bruzzone et al., 2003; Pelegrin and Surprenant, 2006). Panx1 is ubiquitously expressed in mammalian tissues and is elevated in the central nervous system in the CNS and skeletal muscle (Cea et al., 2012, 2014). In humans and mice, high levels of *Panx1* gene transcripts have been found in the nervous system, heart, gonads, kidney, and skeletal muscles (Baranova et al., 2004; Jorquera et al., 2013; Riquelme et al., 2013; Cea et al., 2014; Langlois et al., 2014; Pham et al., 2018). Panx1 forms membrane hemichannels that, upon activation, mediate small molecule communication with the extracellular environment. As a proven conduit for paracrine signaling, Panx1 has been associated with calcium wave propagation, and nucleotide release, including pre-apoptotic “Find Me” signaling, which is transduced via activation of P2 purinergic receptors (Iglesias et al., 2008; Chekeni et al., 2010; Makarenkova et al., 2018). In this context, direct interaction between the P2X7 receptors and Panx1 has been shown to be necessary for ATP release from the cell (Locovei et al., 2006; Schenk et al., 2008; Qiu and Dahl, 2009). A growing number of studies demonstrate that Panx1-mediated ATP release is involved in essential physiological functions such as vasoregulation (Lohman et al., 2012; Gaynullina et al., 2015), and long-range Ca^2+^ wave propagation in endothelial cells (Iglesias et al., 2008) and astrocytes (Iglesias et al., 2009). Panx1-mediated ATP release is essential for paracrine or autocrine activation of purinergic receptors in many tissues, including brain and skeletal muscles (Bruzzone et al., 2003; Baranova et al., 2004; Prochnow et al., 2012). In skeletal muscle, Panx1 has been implicated in potentiation of myofiber contraction by altering ATP and calcium flux (Riquelme et al., 2013; Cea et al., 2014); however, the role of pannexins in muscle regeneration is not well defined. A recent report suggested that Panx1 is important for primary myoblast differentiation *in vitro* (Langlois et al., 2014); however, the role of Panx1 in muscle regeneration *in vivo* has not been studied, and the mechanism(s) by which it controls myoblast differentiation are unknown.

In this study, we show that Panx1 is expressed both by undifferentiated satellite cells/myoblasts and differentiated skeletal muscle fibers. Genetic ablation of Panx1 or pharmacological perturbation of Panx1 or its downstream signaling partners disrupted myoblast differentiation leading to the formation of smaller myofibers. Loss of Panx1 inhibited cell migration by attenuating cell blebbing, and also inhibited myoblast fusion. These findings are consistent with our previous findings that bleb formation is required for myoblast migration and fusion (Makarenkova et al., 2009). Mechanistic studies show that Panx1 activates the P2 (P2X7) purinergic receptor which initiates a lipid signaling cascade that culminates in the activation of myosin-based contraction of the cortical cytoskeleton which supports migration and fusion through the formation of plasma membrane blebs. In addition, Panx1 is required for the expression of several adhesion molecules and a Disintegrin-like and Metalloprotease domain with Thrombospondin-type 5 motifs (ADAMTS5), which have been implicated in ECM remodeling essential for the myoblast fusion process (Stupka et al., 2013).

This study reveals a novel pathway for regulation of myoblast migration and fusion involving Panx1 activated purinergic signaling, lipid metabolism and ECM remodeling, providing important new insights into the molecular mechanisms that underpin muscle regeneration.

## Materials and Methods

### Mice

In this study we used two *Panx1* null mouse strains: the *Panx1KO/B6* (Genetech Inc.) and the *CMV-Cre/Panx1 strain*. The *Panx1KO/B6* (Qu et al., 2011) was backcrossed to the *C57BL/6* background (see Supplementary Methods) for 11 generations. The *Panx1* null strain *CMV-Cre/Panx1^*fl/fl*^* carries a floxed Panx1 allele that was activated with CMV-Cre to create a global knockout. This strain also carries a “passenger” *Casp11* mutation (Dvoriantchikova et al., 2012). Caspase11 is induced in macrophages in response to injury or exposure to bacterial metabolites but is not detectable in homeostatic conditions in tissues such as muscle (Kang et al., 2000; Py et al., 2014). The *Casp11^–/–^* [*B6.129S4(D2)-Casp4^*tm1Yuan*^/J*] mouse strain in the *C57BL/6* background (Jackson Labs) was used as a control for any effects that might be mediated by loss of *Casp11* in the CMV-Cre/Panx1 strain. Wildtype *C57BL/6* mice were used as an additional control.

Mice were housed under standard conditions of temperature and humidity, with a 12-h light/dark cycle and free access to food and water. All experiments were performed in compliance with the Guidelines for the Care and Use of Laboratory Animals published by the United States National Institutes of Health (NIH Publication No. 85-23, revised 1996) and were pre-approved by the Scripps Research Institute Animal Care and Use Committee.

### Myoblast Isolation and Growth

Primary myoblasts were isolated from limb muscles of 3-week-old mice. Satellite cells were isolated using gentleMACS^TM^ Octo Dissociator and either the MACS Microbid satellite cell isolation kit (Miltenyl Biotech, CA, United States) or Fluorescence Activated cell sorting (FACS) as described previously (Sacco et al., 2010; Meech et al., 2011). Cells were grown on collagen-coated plates or chamber slides in growth medium (GM: 1:1 Ham’s F10/DMEM, supplemented with 20% FBS, and 2.5 ng/ml of basic FGF; Rando and Blau, 1994; Makarenkova et al., 2009). To induce myoblast differentiation, the GM was replaced with a differentiation medium (DM: DMEM supplemented with 2% horse serum).

### Injection of Myotoxins (Notexin, Cardiotoxin) and Muscle Regeneration Analysis

Tibialis anterior (TA) muscles of anesthetized 3-month-old Panx1-null and wild-type mice were injected with cardiotoxin (CTX, 100 ng) to induce muscle injury, or with saline (control) as described previously (Sacco et al., 2010; Meech et al., 2011). Muscles were dissected at 2–10 days post-injury, fixed with buffered 4% paraformaldehyde (PFA). Paraffin sections prepared from the muscle midsection were stained with Hematoxylin and Eosin (H&E), and Masson’s Trichrome to detect collagen deposition, and imaged using a Leica scanner.

### Gene Expression Analysis

For gene expression analysis, RNA was extracted using Trizol or Zymo Direct-Zol RNA Kit (Zymo Research Corporation, Irvine, CA, United States) either from dissected TA tissues or from myoblast cultures. The amount of total RNA was estimated using a Nanodrop ND-1000 spectrophotometer and RNA purity and integrity (RIN) was assessed using a Bioanalyzer-2100 device (Agilent Technologies, Inc., Santa Clara, CA, United States). RNAs with RIN between 9 and 10 were used in all experiments. RNA was reverse transcribed to cDNA using the RT^2^ First Strand Kit (#330404, Qiagen, Valencia, CA, United States). The Mouse ECM & Adhesion Molecules RT^2^ Profiler PCR Array (PAMM-013Z; Qiagen) and RT^2^ SYBR Green ROX qPCR Mastermix (#330522; Qiagen, Carlsbad, CA, United States) were used to measure expression levels of 84 individual genes involved in cell–cell and cell–matrix interactions. All other gene-specific primers were obtained from Qiagen. Quantitative real-time PCR was performed using an ABI 7300 Real-Time PCR System (Applied Biosystems, Life Technologies, Carlsbad, CA, United States). Statistically significant differences in threshold cycle (Ct) for each reaction were determined using the RT^2^ Profiler PCR Array Data Analysis software v3.5 (Qiagen). Gene expression differences were filtered for >1.5-fold change and significance *P* < 0.05. Genes with highly variable Ct values were excluded from the final analysis. Reference genes for normalization of real-time PCR data were b-actin (ACTB), β-2 microglobulin (B2M), and glyceraldehyde-3-phosphate dehydrogenase (GAPDH).

### Immunocytochemistry

Myoblast cultures were fixed with 2% PFA in PBS pH 7.5 for 20 min, permeabilized in Tris–buffered Saline with 0.1% Tween 20 (TBST), and blocked with 5% goat serum. Cells were immunostained with primary antibodies overnight at 4°C, followed by staining with appropriate secondary antibodies (antibodies are detailed in Supplementary Methods) and nuclear staining with DAPI. Images were taken using Zeiss LSM 710 laser scanning confocal microscope (LSCM).

### Antibodies

Primary antibodies used: affinity purified rabbit Panx1 antibody CT-395 (Px-34; Penuela et al., 2007) was kindly provided by Dr. D. W. Laird (University of Western Ontario, Canada), rabbit polyclonal anti-human Panx1 antibodies (Chemicon, Inc.); mouse monoclonal anti-Myogenin (clone F5D, BD Bioscience Pharmingen, San Diego, CA, United States), mouse monoclonal anti-MyoD (clone MoAb5.8A, BD PharMingen, San Diego, CA, United States), pan myosin heavy chain (MyHC; A4.1025 Developmental Studies Hybridoma Bank, Iowa City, IA, United States), and mouse monoclonal anti-sarcomere myosin (clone MF20, Developmental Studies Hybridoma Bank, Iowa City, IA, United States), antibodies.

### Immunoblotting

Total protein was prepared from primary myoblast cultures using Nonidet P-40 lysis buffer and sonicated. Equal aliquots of protein were resolved by SDS-PAGE using the 4% to 12% Bis-Tris gels (NuPAGE; Invitrogen), transferred to polyvinylidene difluoride membrane, and probed with polyclonal antibodies to Panx1 (Chemicon) and to β-actin, β-tubulin, or GAPDH. Bands were visualized by chemiluminescence using X-ray film exposure and quantified by densitometry. Each experiment was performed in duplicate.

### Single Myofibre Isolation and Culture

Single muscle fibers were isolated from the extensor digitiorum longus (EDL) muscle of 12-week old male C57Bl6 as described previously (Otto et al., 2008, 2011). Briefly, the EDL was dissected with both tendons intact and myofibers liberated by digestion with (0.2%) type I collagenase (Sigma) in Dulbecco modified eagle medium (DMEM) at 37°C under 5% CO_2_. Using tapered glass pipettes, single fibers were plated in single fiber culture medium (SFCM, DMEM supplemented with 10% horse serum, 0.5% chick embryo extract, and 1% penicillin/streptomycin).

### Time-Lapse Light Microscopy and Electron Microscopy

Following 24 h of culture, myofibers were monitored using a phase contrast time-lapse microscope system. Myofibers grown in chamber sides were imaged in an environmental chamber maintained at 37°C and supplemented with 5% CO_2_. Time-lapse video was taken at a rate of 1 frame every 15 min over 24 h for satellite cells using a 10X objective, a previously well-studied time course for satellite cell migration following single fiber isolation (Otto et al., 2008, 2011). All video analysis was carried out using freeware package ImageJ (version 1.49 m). Satellite cells were individually manually tracked using the MTrackJ plugin on ImageJ.

### Electron Microscopy

For electron microscopy, single myofibers were fixed following 48 h in standard culture with 4% PFA for 15 min, and then processed.

Fixed myofibers were dehydrated through 30, 50, 70, 80, 90, and 100% ethanol solution series (15 min for each step) and transferred to a critical point drier (Balzers CPD 030, using liquid carbon dioxide) thereafter. Dried myofibers were then carefully transferred to scanning electron microscopy (SEM) chucks using micro-forceps under a light microscope. Myofibers were then gold-coated using an Edwards S150B sputter-coater. Coated myofibers were then visualized under an FEI 600F SEM using the accompanying analysis software for image collection.

### Mathematical Data Analysis

Mathematical models of particle motion are able to provide us with characterizations, which can be compared with data in order to predict how cells are moving (Woolley et al., 2013, 2014). Although we have shown that the shape of the cell and its blebs are critical to its migration properties (Woolley et al., 2015a,b), here, we simply characterize the motion characteristics in terms of the migration data. Specifically, we model the cells as random walkers on a straight, cylindrical fiber. Previously (Collins-Hooper et al., 2012), we have shown that, under this assumption, we can separate the cellular motion into two components; (i) a purely angular motion of the cell, around the muscle fiber and (ii) a purely longitudinal motion, along the axis of the fiber. We primarily focus of this longitudinal motion as it is the most crucial mode of movement.

Due to the probabilistic nature of the problem, we cannot specify exactly where the cell will be at all times in the future, however, we can predict the probability distribution of the cell locations. We define this probability distribution to be *p*(*x*,*t*), which represents the probability of finding a cell at a position *x* (measured along the fiber) at a given time *t*. The distribution satisfies the standard diffusion equation in one dimension (Belmonte-Beitia et al., 2013),

$$\frac{\partial p}{\partial t}=D\,\frac{\partial^{2}p}{\partial x^{2}},p\,\left(x,0\right)=0,p\,\left(\infty ,t\right)=0.$$

This equation simply encapsulates the idea that the cells are moving randomly, all of the cell’s positions are normalized to start at zero and the fiber has no boundaries. The *D* parameter is a positive constant that measures that rate at which the cells spread out from the normalized origin. From this equation we derive that the mean squared displacement of the cells is proportional to time,

$$\left\langle x^{2}\right\rangle =2\,D\,t.$$

Thus, taking the trajectory data we normalize the start position of all trajectories to zero and rotate all trajectories such that the fiber lies along the *x*-axis. The mean square distance moved by the cell along the fiber is calculated, and a straight line is fitted to this derived statistic. A goodness of fit *R*^2^ value is also computed to express how well the straight line fits the data. The closer the *R*^2^ value is to one, the closer the movement of the cells is to a memoryless, random diffusion.

### Pharmacological Treatments

#### Carbenoxolone Disodium

Primary myoblasts were treated with carbenoxolone (CBX, Sigma-Aldrich # C4790) or vehicle, for 24 h prior and then induced to differentiate in low serum medium also containing CBX or vehicle (control). CBX blocks pannexin and connexin channels at 100 μM, but has high selectivity for pannexin channels at 10–50 μM (Bruzzone et al., 2005). Varying CBX concentrations from 10 to 100 μM were tested and 25 μM was found to be optimal for pannexin inhibition. Myoblast cultures treated with 25 μM CBX in DM were fixed at different time points 6, 12, 24, and 72 h and processed for immunostaining.

#### Purinergic Receptor Inhibitor A740003

A740003 is a potent, selective and competitive P2X7 receptor antagonist that displays selectivity to P2X7 receptor up to a concentration of 100 μM. A740003 (Tocris, # *3701*) at 50–100 nM or vehicle (dimethylsulfoxide) was applied to myoblasts 24 h prior to induction of differentiation. GM was replaced with DM containing A740003 or vehicle and cells were fixed at different time points for immunostaining.

#### Apyrase

To disrupt ATP signaling, cultures were treated with the ATP hydrolyzing enzyme apyrase (2.5 U/ml, Sigma, added to GM) or vehicle 24 h prior to the induction of differentiation. GM was then replaced with DM also containing apyrase or vehicle (control).

To study the role of lipid signaling, single muscle fibers were cultured with the following drugs 24 h post isolation: Butanol (1% v/v Fisher # A383-1), that inhibits the action of PLD in the formation of phosphatidic acid; VPC *32183* (1 μM Avanti Polar Lipids # 857340), a selective inhibitor of the LPA_1_ and LPA_3_ receptors; ML-7 (50 μM Sigma # I2764), a selective myosin light chain kinase inhibitor; Y-27632 (10 μM Sigma # Y0503), a potent inhibitor of Rho-associated kinase; and LPA (10 μM Sigma # L7260).

### Myoblast Fusion Index

To determine the fusion index, cultures were stained with the MF20 antibody and the numbers of nuclei were counted in MyHC+ cells. Fusion index was calculated as the number of nuclei in multinucleated MyHC+ cells (≥2 nuclei) as a proportion of total nuclei. Three independent experiments were performed for each condition; approximately 500 nuclei were counted per condition.

### Quantification of Pericellular Matrix Area

The erythrocyte exclusion assay was performed as previously described (Hattori et al., 2011; Stupka et al., 2013). Briefly, Panx1 and WT primary myoblasts were plated into two-well chamber slides and cultured for 24–48 h. The culture medium was replaced by freshly prepared erythrocyte suspension (1 × 10^7^ erythrocytes/ml) in serum-free DMEM. After erythrocytes had settled to the bottom, images (20–30 per experimental group) were taken with an inverted microscope. In some assays, Streptomyces hyalurolyticus hyaluronidase (H1136, Sigma) was added prior to erythrocyte addition.

### Statistical Analysis

Statistical significance was assessed using unpaired two-tailed Student’s *t*-test and results were considered significant at *P* < 0.05, or *P* < 0.01. Results were expressed as mean ± s.d.m (standard deviation of the mean).

## Results

### Panx1 Is Expressed in Satellite Cells and Is Increased During Myoblast Differentiation

**FIGURE 1:**
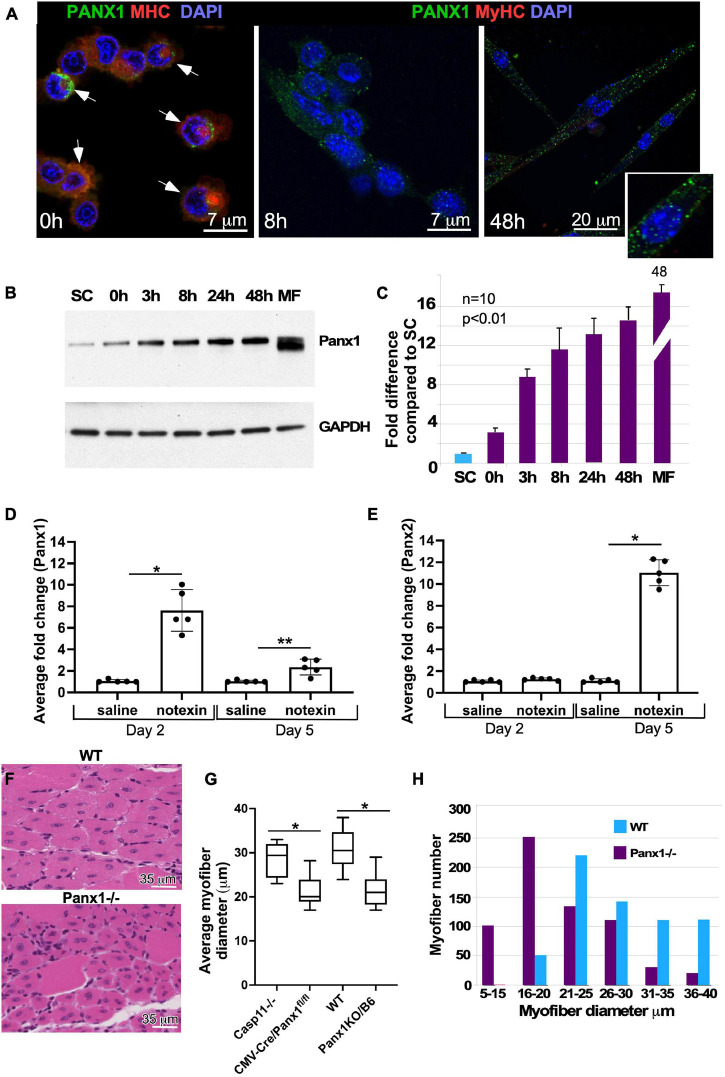
Panx1 expression in primary myoblasts and role of Panx1 in muscle regeneration. **(A)** Migrating myoblasts express Panx1 (green) in the plasma membrane. Panx1 expression levels are increased in myoblasts forming membrane protrusions, i.e., blebs (indicated by white arrows), stained with SMA (red) to visualize blebbing. Panx1 expression (green) is up-regulated in differentiating myoblasts (8 h in DM) and early myotubes (48 h in DM). An inset shows higher magnification of cells maintained 48 h in DM. Nuclei are stained with DAPI (blue). **(B,C)** Panx1 expression increases during myoblast differentiation. **(B)** Western blot showing that undifferentiated freshly isolated SCs show the lowest level of Panx1; Panx1 expression increases during myoblast differentiation (0, 3, 8, 24, and 48 h in DM), peaking in adult myofibers (MF). **(C)** Quantification of western blotting data (*n* = 3 independent experiments); *P* < 0.05. **(D,E)** Quantitative RT-PCR analysis of *Panx1* **(D)** and *Panx2*. *p < 0.0001; **p < 0.001. **(E)** Expression at 2 or 5 days after CTX injection into the TA muscle. Gene expression data from the CTX-treated limb was normalized to that from the vehicle (saline)-treated contralateral limb; *n* = 3 mice per time point, *P* < 0.01, *p < 0.0001. **(F)** Hematoxylin and eosin-stained transverse sections of TA muscle from *WT* and *Panx1^–/–^* (*CMV-CrePanx1^*fl/fl*^*) mice 10 days after muscle injury. Newly formed (centronucleated) Panx1*^–/–^* myofibers are smaller in diameter than newly formed WT myofibers. **(G)** Quantification of myofiber sizes shows that myofibers from both *Panx1^–/–^* mouse strains (*CMV-CrePanx1^*fl/fl*^* and Panx1KO/B6) are on average ∼30% smaller than myofibers from *Casp11^–/–^* and *WT* control mice. *p < 0.0001. **(H)** Histogram demonstrating the distribution of regenerated myofiber sizes in *Panx1^–/–^* (*CMV-CrePanx1^*fl/fl*^*) and control mice. Minimal myofiber diameters were measured in transverse sections of TA muscle from three mice for each genotype. *n* = 647 fibers for *Panx1^–/–^* and 636 for *WT*.

Panx-1 was previously shown to be expressed in skeletal muscle (Langlois et al., 2014); however, its role during muscle differentiation and regeneration remains largely unknown. We examined the temporal pattern of PANX1 protein expression during myoblast differentiation using immunocytochemical, quantitative Western blot and gene expression analyses of primary cultures. Panx1 was expressed in isolated satellite cells (Figure 1A) and was increased rapidly at both mRNA and protein levels after the induction of myoblast differentiation (Figures 1A–C).

### The Role of Panx1 in Muscle Development and Regeneration

Muscle development and regeneration in the absence of Panx1 have not been previously studied. Basic morphometric analysis of muscle groups (TA, gastrocnemius, and soleus) in *WT* vs. *Panx1^–/–^* mice showed that neither postnatal nor adult *Panx1^–/–^* mice have any significant change in the size of limb muscles (data not shown). These results suggest that loss of Panx1 does not significantly affect muscle development and/or postnatal muscle growth.

To determine whether Panx1 may play a role in regeneration, we first examined the dynamics of pannexin expression during muscle regeneration *in vivo*. The TA muscle of WT mice was injured with notexin and changes in the levels of Panx 1–3 transcripts post-injury were assessed by qRT PCR. The Panx1 transcript levels were higher than those of the *Panx2* and *Panx3* genes at all stages (data not shown). On day 2 after notexin-injury (muscle inflammation/early regeneration phase), there was a 7.6 ± 1.9-fold increase in the amount of Panx1 transcript in injured vs. saline-injected control TA muscle (Figure 1D), while the amount of Panx2 transcript remained unchanged (Figure 1E). On day 5 after injury (late differentiation phase), Panx1 levels decreased relative to the day 2 peak level and was 2.4 ± 0.7 (Figure 1D). In contrast, Panx2 levels at this time point increased 11 ± 1.2-fold (Figure 1E). These findings suggest that these two pannexins may play different roles in the succession of injury-induced muscle repair phases. As expected (Langlois et al., 2014), the Panx3 mRNA level during muscle regeneration was very low and in some samples undetectable (data not shown).

We next investigated the role of Panx1 in muscle regeneration using two different Panx1 null models: *CMV-Cre/Panx1^*fl/fl*^* mice for which *Casp11^–/–^* mice served as the relevant control, and Panx1KO/B6 mice with *WT* mice as the control. TA muscles were injured with notexin and regeneration studied 10 days later. All mice showed regeneration as evidenced by appearance of new myofibers with centrally located nuclei (Figure 1F). However, the newly regenerated myofibers in both *Panx1^–/–^* models were significantly smaller in diameter when compared to those of the control strains (Figure 1G). In addition, we found that regenerated TA muscles of *Panx1^–/–^* mice were depleted for large myofibers and enriched in smaller myofibers, as compared to wild-type TA muscles (Figure 1H).

### Genetic Ablation of Panx1 Affects Myotube Formation

**FIGURE 2:**
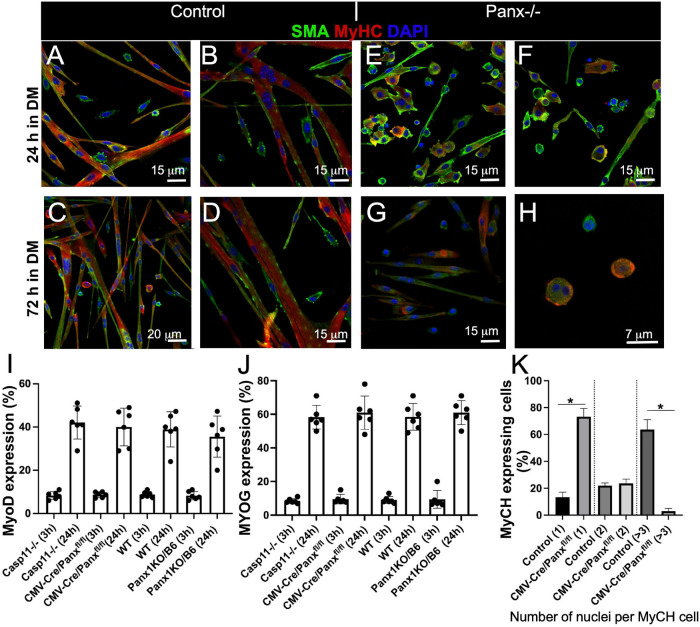
Loss of Panx1 function impairs myoblast fusion but not activation of the molecular differentiation program. WT myoblasts form myotubes after 24 h in DM **(A,B)**, while the majority of Panx1^–/–^ myoblasts remain unfused **(C,D)**. After 72 h in DM, WT cells form long myotubes and only a few single cells remain **(E,F)**. In contrast *Panx1^–/–^* cultures show fewer myotubes and many more single myoblasts **(G)**. **(H)** Higher magnification of cultures shown in **(G)** shows that even single myoblasts in these Panx1^–/–^ cultures express MyHC (red). In all panesl SMA (green), MyHC (red), DAPI (blue). Quantification of MyoD **(I)** and MYOG **(J)** expressing cells in *WT* and *Casp11^–/–^* controls and *Panx1^–/–^* (*CMV-CrePanx1^*fl/fl*^* and Panx1KO/b6) myoblasts 3 and 24 h after induction of differentiation. MyoD and MYOG expression is calculated as the percentage of MyoD and MYOG positive nuclei. **(K)** Fusion index is calculated as the percentage of MyHC-positive cells containing one, two, or ≥3 nuclei at 48 h after induction of differentiation. Data are presented as mean ± standard deviation. *n* = 3 independent experiments. *P < 0.05 compared with controls.

The reduction in myofibre size in regenerating *Panx1^–/–^* muscle suggested a possible myoblast fusion defect. We thus compared differentiation of *Panx1^–/–^* (both *Panx1-null* strains) and control (*Casp11^–/–^* and/or *WT*) myoblasts in culture. Control myoblasts cultured in DM formed numerous multinucleated myotubes (Figures 2A,B and Supplementary Figure 1A) and by 72 h the cultures were almost free of unfused myoblasts (Figures 2C,D and Supplementary Figure 1A). In contrast, *Panx1^–/–^* myoblasts developed very few multinucleated cells over 72 h in DM (Figures 2E–H and Supplementary Figure 1B). Analysis of *Casp11^–/–^* myoblast differentiation showed that they differentiated normally and did not differ morphologically from WT myoblasts. Both differentiating control and *Panx1^–/–^* cultures expressed MyHC protein (Figures 2A–H).

The process of myoblast differentiation is tightly controlled by myogenic regulatory factors (MRFs). Cultured myoblasts normally express MyoD in GM and initiate expression of myogenin as early as 1–3 h after induction of differentiation (Makarenkova et al., 2009). There was no difference in the number of MyoD expressing cells in *Panx1^–/–^* relative to control myoblasts at 3 and 24 h-hours post induction of differentiation (Figure 2I). Moreover, the proportion of myogenin (MYOG)-positive nuclei in *Panx1^–/–^* and control myoblast cultures 3 and 24 h after induction of differentiation was not significantly different (Figure 2J). The time to induction of MyHC and percentage of MyHC expressing cells were also similar in both cultures (Figures 2A–H). However, relative to control (WT and *Casp11^–/–^)* myoblasts, *Panx1^–/–^* myoblasts formed significantly fewer multi-nucleated (3 or more nuclei per cell) MyHC positive myotubes (Figure 2K and Supplementary Figure 1C). Interestingly, unfused myoblasts in *Panx1^–/–^* cultures also expressed MyHC despite the impaired myotube formation (Figures 2G,H). In summary, our results suggest that activation of the myogenic differentiation program is not affected in *Panx1^–/–^* myoblasts. This finding corroborates previous reports that myoblast fusion is not required for the expression of MyHC (Devlin and Emerson, 1978, 1979).

### Genetic Ablation of Panx1 Affects Myoblast Blebbing and Migration

**FIGURE 3:**
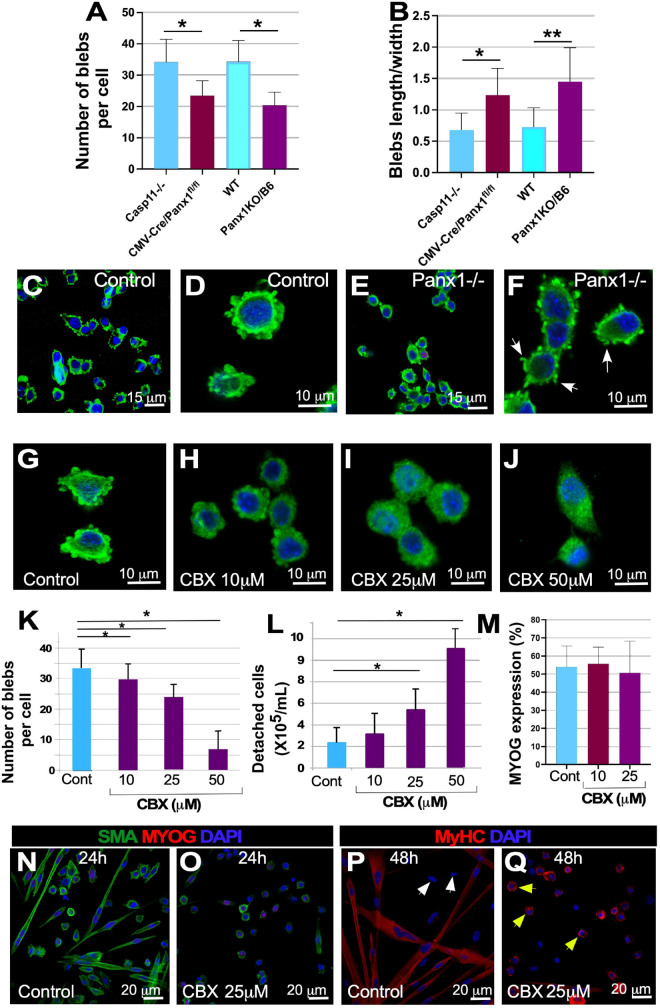
Panx1 signaling regulates myoblast blebbing, migration, myoblast fusion and myofiber formation *Panx1^–/–^* myoblasts have fewer surface blebs **(A)** and altered bleb shape **(B)**, as shown by comparison of bleb length/width ratio in *control* and *Panx1^–/–^* myoblasts. Data are presented as mean ± standard deviation. *n* = 3 independent experiments. *P < 0.05. The appearance of blebs in control **(C,D)** and *Panx1^–/–^* myoblasts stained with αSMA **(E,F)**. **(G–J)** Blocking Panx1 signaling in myoblast cultures with carbenoxolone (CBX) decreases blebbing: myoblasts treated with vehicle **(G)**, 10 μm **(H)**, 25 μm **(I)**, 50 μm **(J)** of CBX for 24 h. Quantification of bleb numbers in control and CBX treated myoblasts **(K)**. **(L)** High-dose CBX treatment decreases cell adhesion and induces cell detachment in plate washing assays. Analysis of the differentiation program in CBX treated myoblasts: **(M–Q)**: MYOG expression was calculated as the percentage of MYOG-positive nuclei **(M)**. **(N–Q)** Cultures were treated with vehicle **(N,P)**. and 25 μm of CBX in DM **(O,Q)** and MYOG **(N,O)** and MyHC **(P,Q)** expression was assessed by immunostaining. MyHC expression was found in the majority of cells in the vehicle **(P)** and CBX-treated **(R)** cultures. Single migrating cells in control cultures (**P**, white arrows) did not express MyHC, while almost all single cells in CBX treated cultures expressed MyHC (**Q**, yellow arrows). Myotube formation was inhibited in CBX-treated **(O,Q)**, compared to control **(N,P)** cultures. Data in **(K–M)** is presented as mean ± standard deviation. *n* = 3 independent experiments. *P < 0.05. **P < 0.01.

To form myofibers, differentiating myoblasts must migrate and establish stable cell-cell contacts (Asakura et al., 2001). Real-time *in vivo* imaging has revealed rapid migration of myoblasts to the site of myofiber damage *in vivo* (Ishido and Kasuga, 2011, 2012; Webster et al., 2016). Several research groups, including ours, reported that rapid movement of myoblasts requires formation of dynamic membrane blebs (Charras and Paluch, 2008; Makarenkova et al., 2009; Otto et al., 2011), as they utilize an amoeboid-based rather than lamellipodia-mediated propulsion mechanism (Otto et al., 2011). Morphometric analysis showed that cultured primary Panx1^–/–^ myoblasts had fewer blebs than control myoblasts (Figure 3A and Supplementary Figure 1, compare A and B). Analysis of *Panx1^–/–^* myoblasts showed that they were significantly less migratory and stayed clustered, in contrast to Control (*WT* and *Casp11^–/–^)* myoblasts that readily spread throughout the dish (Figures 3C,E). Moreover, while control myoblasts had wide, rounded surface blebs, the blebs on Panx1^–/–^ myoblasts were thinner. The average length-to-width ratio of *Panx1^–/–^* myoblast blebs was 2-fold less than that of controls (1.23 ± 0.42 for *CMV-CrePanx1^*fl/fl*^* vs. 0.79 ± 0.18 for *Casp11^–/–^* and 1.45 ± 0.54 for *Panx1KO/B6* vs. for *WT* myoblasts, *p* > 0.001), (Figures 3B,D,F).

We next tested whether pharmacological blockade of Panx1 channels in culture could phenocopy Panx1 genetic ablation. Carbexonolone (CBX) inhibits ATP release through pannexin channels (Hayoz et al., 2012; Xia et al., 2012) and has high specificity for Panx1 channels at <100 μM (Bao et al., 2012). Undifferentiated wildtype myoblasts were exposed to CBX (10, 25, and 50 μM) for 24 h prior to induction of differentiation and then transferred into DM also containing CBX. Myoblast treatment with CBX for 24 h produced significant changes in the morphology and number of surface blebs. With increasing CBX concentration, the number of surface blebs per cell decreased significantly after 10 μM CBX applicationammount of protein; at 25–50 μM CBX myoblasts retained only a few small protrusions (Figures 3G–K).

The morphological changes induced by CBX negatively correlated with adhesion of treated myoblasts to the collagen substrate, causing cells to detach in plate-washing assays more readily (Figure 3L and Supplementary Methods). Thus, 50 μM CBX treatment caused a 5-fold increase in myoblast detachment relative to control (Figure 3L). In contrast, treatment with 10–25 μM of CBX did not induce significant myoblast detachment and we used these drug concentrations in all further experiments. After 24 h exposure to 10 and 25 μM CBX, myoblasts were induced to differentiate in DM also containing CBX, and expression of MYOG and MyHC was assessed by immunocytochemistry (Figures 3M–Q). Similar to *Panx1^–/–^* myoblasts, the number of MYOG expressing cells remained unchanged in CBX treated myoblasts (Figures 3M–O), implying that the core transcriptional differentiation program remained unaltered. The induction of MyHC protein expression also appeared unaffected in control and CBX treated cultures (Figures 3P,Q and Supplementary Figures 2A,B). However, unlike control myoblasts (Figures 3N,P), myoblasts treated with CBX failed to elongate and showed almost no fusion (Figures 3O,Q). The similarities in differentiation deficiencies of CBX-treated and *Panx1^–/–^* cells, suggest that Panx1 and ATP mediated signaling controls myoblast migration and fusion.

### ATP Signaling Controls Blebbing and Migration via Membrane Derived Lipid Intermediates

**FIGURE 4:**
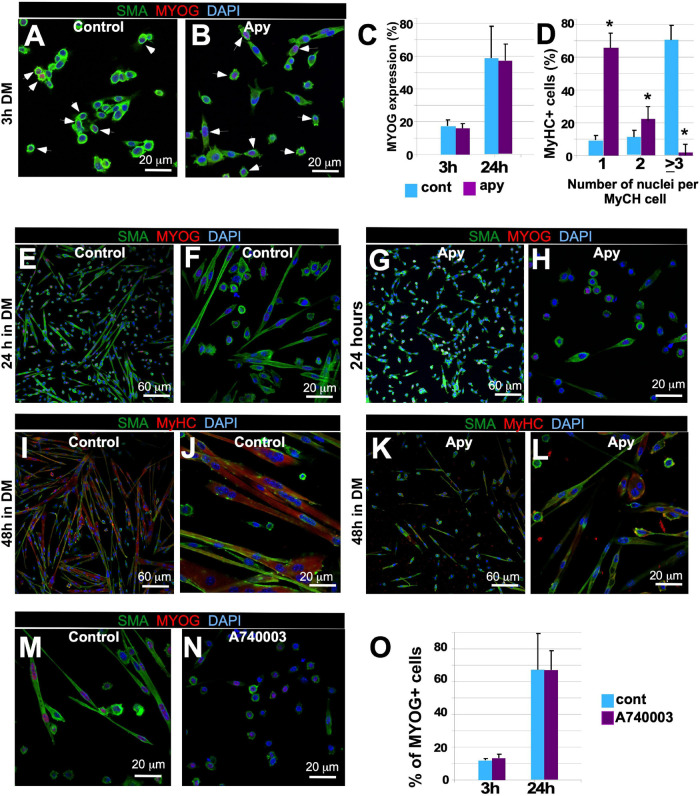
Disruption of ATP or P2X7 receptor signaling inhibits myoblast fusion but not the molecular differentiation program. **(A–L)** Myoblast cultures were treated with ATP hydrolyzing enzyme apyrase (2.5 U/ml) or vehicle 1 day prior to the induction of differentiation. GM was then replaced with the DM also containing apyrase or vehicle. Myogenin (MYOG) expression (**A**-control, **B**-apyrase, white arrows) was assessed by immunostaining; MYOG – red, SMA – green, DAPI – blue, scale bar is 20 μm. The percentage of MYOG expressing cells was quantified **(C)** 3 h after induction of differentiation with and without apyrase treatment (compare **I**, **J** with **K**, **L**). **(D)** The percentage of MyHC-positive cells containing one, two, or ≥3 nuclei was quantified after 48 h of differentiation. Data are presented as mean ± standard deviation. *N* = 3 independent experiments *p < 0.0001. Control (vehicle-treated; **E,F** – 24 h in DM; **I,J** – 48 h in DM) and apyrase treated (**G,H** – 24 h in DM; **K,L** – 48 h in DM) primary myoblast cultures. Myoblast fusion is impaired in apyrase treated cultures (**E–H**; SMA – green; MYOG – red, and DAPI – blue; **I–L**; SMA – green, MyHC – red, and DAPI – blue). Scale bars: in **(E,G,I,K)** – 50 μm; in **(F,H,J,L)** – 20 μm. The A740003 treatment inhibited myotube formation **(N)** relative to vehicle **(M)** treated cultures; activation of the differentiation program as assessed by quantification of the percentage of MYOG expressing cells **(O)** was unaffected at 3 and 24 h after induction of differentiation. In **(M,N)**, SMA – green, MYOG – red, and DAPI – blue. Scale bars are 20 μm.

Next, we examined whether signaling downstream of Panx1, particularly release of intracellular ATP, is involved in myoblast migration and fusion. We treated myoblasts with apyrase, an enzyme that catalyzes the breakdown of ATP into AMP and inorganic phosphate. Apyrase treatment did not alter the timing of MYOG expression or the number of MYOG-expressing cells (Figures 4A,B, white arrows, C), but dramatically inhibited myotube formation (Figures 4D–L). After 24 h in DM, apyrase treated myoblasts elongated but did not form myotubes (Figure 4, compare E, F with G, H) and after 48 h the percentage of myotubes with 3 or more nuclei was much lower than in control cultures (Figure 4D, compare I, J with K, L). This effect phenocopied *Panx1^–/–^* and CBX-treated myoblasts (see Figures 2, 3). Thus, both ablation of Panx1 and pharmacological blockade of ATP lead to altered cell shape, migration, and fusion properties, suggesting that Panx1 mediates these events through ATP signaling.

Extracellular ATP is known to transmit signals by activating purinergic ionotropic P2X or metabotropic P2Y receptors (Wilkinson et al., 1993; Meyer et al., 1999; Ryten et al., 2004; Martinello et al., 2011); involvement of P2X receptors in C2C12 myoblast differentiation was documented previously (Araya et al., 2004). To test whether purinergic signaling via P2X7 is involved in myoblast migration and differentiation, we blocked this receptor in WT primary myoblasts with P2X7 receptor antagonist A740003. A740003 treatment inhibited myotube formation relative to control (Figures 4M,N), while again MYOG expression was unaffected (Figure 4O). These data suggest an important role for P2X7-mediated signaling in myotube formation.

**FIGURE 5:**
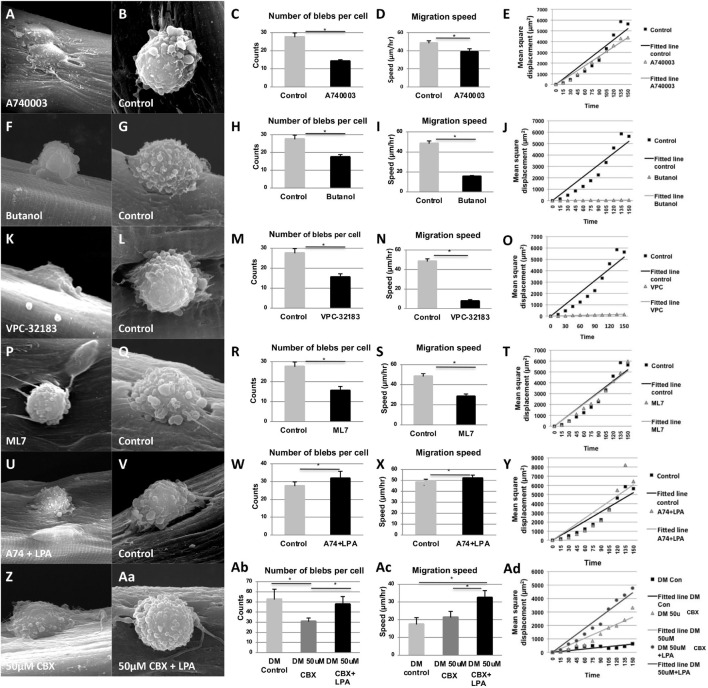
Panx1 regulates a lipid-based signaling pathway that controls bleb formation and satellite cell migration on single myofibers. Quantification of SEM images shows a reduction in the number of blebs per cell, reduced migration speed, but no change in the directionality of movement (plotted as mean square displacement over time) after treatment with A740003 **(A–E)**, Butanol **(F–J)**, VPC-32183 **(K–O)**, and ML7 **(O–T)**. The inhibitory effect of A740003 **(U–Y)** and CBX on blebbing and migration were **(Z–Ad)** rescued by LPA. Treatment with LPA resulted in a significantly higher migration speed than the control cells **(X,Ac)**. For movement directionality plots, control data are represented by black squares and treatment data by gray triangles. Black and gray lines represent best fit curves; straight fit curves indicate random migration. *p < 0.05.

We next examined the effect of inhibiting the P2X7 channel on cell blebbing and migration using single myofiber cultures where individual satellite cells migrate along the myofibers. Treatment of fiber associated-satellite cells with A740003 significantly reduced bleb formation and migration speed (Figures 5A–E). Lipid signaling mediated by LPA was shown to be a major determinant in bleb formation in a variety of cell types (Hagmann et al., 1999; Jia et al., 2006). Moreover, P2X7 receptors activate a signaling pathway that induces Phospholipase D (PLD) to convert phosphatidylcholine to phosphatidic acid (PA) which in turn is transformed into lysophosphatidic acid (LPA) by Phospholipase A_2_ (Panupinthu et al., 2007). The hydrolytic reaction mediated by PLD requires water and is inhibited by primary but not tertiary alcohols. To assess whether P2X7 may control blebbing through (L)PA production, we repressed PLD activity by addition of 1-butanol to satellite cells on their native substrate and found that it resulted in a significant decrease in the number of blebs displayed on the cell surface, as well as a reduction in migration speed (Figures 5F–J). In a control experiment, an identical concentration of tertiary butanol had no effect on either parameter (Figures 5G–J). These data indicate that P2X7-facilitated blebbing and migration is reliant on PLD activity to form PA. The action of lipids to induce signaling cascades is heavily dependent on the conversion of PA into LPA by phospholipase A_2_, which in turn activates the LPA receptor, a G-coupled protein that controls a diverse activity including cell migration. We examined whether LPA activity was important in blebbing and satellite cell migration using VPC-32182, a selective LPA1 and LPA3 receptor antagonist. Treatment with VPC-32182 dramatically reduced bleb formation and rate of satellite cell migration (Figures 5K–O), showing a role for LPA activity in these processes. LPA receptors can activate Rho, which ultimately leads to myosin light chain phosphorylation hence controlling actin/myosin-based contraction which may drive bleb dynamics. We found that inhibition of myosin light chain kinase with a ML7 inhibitor reduced satellite cell blebbing and migration speed (Figures 5P–T).

To show a more direct link between Panx1/P2X7-mediated ATP signaling and the activity of the LPA receptor in controlling blebbing and migration, we inhibited Panx1 channels with CBX or P2X7 receptors with A740003, and simultaneously introduced LPA. The inhibitory effect of both CBX and A740003 on bleb formation and migration was rescued by LPA (Figures 5U–Y for A740003 and Z-Ac for CBX).

Finally, we used mathematical modeling to examine whether interfering with the Panx1-mediated lipid based signaling pathway in myoblasts influenced not only migration speed but also directionality. We have previously demonstrated that satellite cells from young mice move in a random, directionless manner on the myofiber that contrasts the behavior of cells from old mice, which show directionality (Otto et al., 2011). Plotting the mean square displacement over time showed that although A740003, Butanol, VPC-32183 and ML7 treatments all decreased migration speed of young myoblasts on single myofiber, they still moved in a random manner indicated by a straight-line plot (Figures 5E,J,O,T). Furthermore, rescue of the migration deficit induced by A740003 or CBX with LPA did not alter the migration mode (Figures 5Z, Aa–Ad).

### Inhibition of Panx1 and Its Downstream Target Molecules Impedes Myoblast Fusion

**FIGURE 6:**
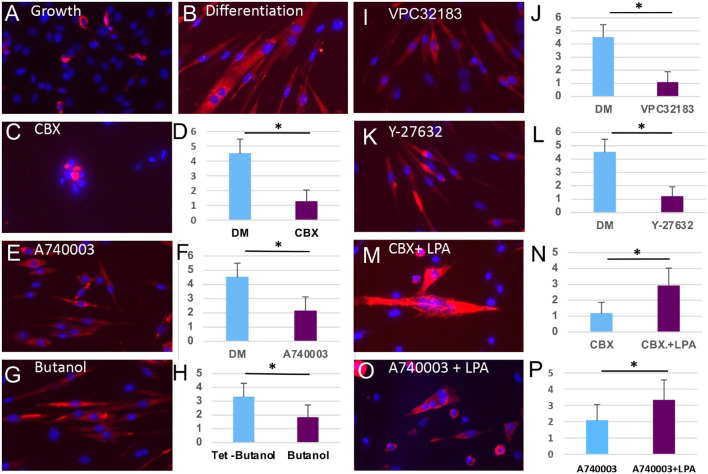
Inhibition of multiple steps of the Panx1 signaling cascade blocs fusion of myoblasts but not the molecular differentiation program. **(A)** Primary myoblasts cultured in GM do not form myotubes and express little MyHC (red). **(B)** Culture of primary myoblasts for 72 h in DM induces the formation of MyHC-expressing myotubes. **(C–L)** Treatment with CBX **(C,D)**, A740003 **(E,F)**, Butanol **(G,H)**, VPC32183 **(I,J)**, and 27632-Y **(K,L)** all inhibit myotube formation (presented as an average number of nuclei per cells) but not the expression of MyHC. The inhibitory actions of CBX **(M,N)** or A740003 **(O,P)** are rescued by the action of LPA (*p* < 0.05). *p < 0.05.

The studies above define a signaling pathway activated by Panx1 that regulates bleb formation and migration of satellite cells. Here we determined whether the same signaling components also regulate myoblast fusion. Freshly isolated satellite cells were cultured at high density permitting cell-cell contact and then maintained in GM or DM for 72 h under a range of drug treatment conditions. In the control conditions, cells in GM remained largely mono-nucleated with few cells expressing MyHC (Figure 6A), while cells in DM formed myotubes with an average of 4.5 nuclei and robust MyHC expression (Figure 6B). Addition of Panx1 channel inhibitor CBX or P2X7 channel inhibitor A740003, to high-density cultures in DM inhibited fusion despite the expression of MyHC (Figures 6C–F). Likewise, fusion was attenuated by addition of butanol, LPA receptor inhibitor VPC32183, or ROCK inhibitor Y-27632 (Figures 6G–L). Finally, inhibition of fusion by blockade of either Panx1 or P2X7 receptor could be reversed by LPA addition (Figures 6M–P). Together these results show Panx1 signaling facilitates myoblast fusion through the P2X7 receptor, downstream lipid intermediaries and the ROCK pathway that activates blebbing.

### Microarray Analysis of Cell Adhesion Pathways

Pannexins mediate dynamic adhesive contacts and thus regulate diverse functions including cell alignment, adhesion, migration and fusion (Gorbe et al., 2007; Bao et al., 2012). To test whether loss of Panx1 affects expression of key genes involved in these processes, we isolated Panx1^–/–^ and WT myoblasts from 4-month-old mice by FACS and profiled the expression of 84 genes important for cell-cell and cell-matrix interactions. We found nine genes that were significantly altered (>1.5-fold) in Panx1^–/–^ vs. WT myoblasts (Table 1). Among the genes significantly up-regulated in Panx1^–/–^ vs. WT myoblasts were Cadherin-2 (Cdh2), Integrin-β4 (Itgb4), Collagen VI (Col6a1), and sparc/osteonectin (SPOCK1). Among the genes down-regulated in Panx1^–/–^ cultures, we identified transforming growth factor, beta-induced gene (TGFBI/BIGH3; 3.04-fold change, *p* = 0.008). TGFBI activation has been previously shown to promote myofibril bundling and ECM deposition (Kim and Ingham, 2009). We also found significant downregulation of Elastin Microfibril Interface-Located Protein 1 (Emilin1) that was recently shown to be responsible for the formation of the elastic fibers and anchoring muscle cells to them (Gundry et al., 2009). Multiple Matrix Metalloproteinases (MMPs) were also down-regulated, particularly MMP12 (5.6-fold; *p* = 0.02), and the disintegrin metallopeptidase with thrombospondin type 5 motif gene (Adamts5, 1.79-fold; *p* = 0.01; Table 1).

**TABLE 1:** Genes significantly up-regulated and down-regulated in Panx1^–/–^ myoblasts.

Gene symbol	Fold regulation	*p*-value
Cdh2	1.6565	0.007231
Col6a1	1.7234	0.00339
Itgb4	1.7888	0.016005
Spock1	2.7921	0.000974
Adamts5	–1.7863	0.010698
Emilin1	–2.3793	0.002628
Itgb1	–2.8438	0.047069
Mmp12	–5.5691	0.02004
Tgfbi	–3.0377	0.00831

*Analysis of gene expression in *Panx1^–/–^* myoblasts compared to *WT* myoblasts. Genes significantly up-regulated in *Panx1^–/–^* myoblasts: *Cdh2 –* N-cadherin; *Col6a1* – Collagen, Type VI, Alpha-1, Itgb4 – Integrin Beta 4; Spock1 – Sparc/Osteonectin (encodes testican-1 protein). Genes, expression of which significantly down-regulated in *Panx1^–/–^* myoblasts compared to *WT* myoblasts: *Adamts5 –* a disintegrin and metalloproteinase with thrombospondin motifs, *Emilin1 –* Elastin Microfibril Interfacer-1, Itgb1 – Integrin, Beta-1, MMP12 – Matrix Metallopeptidase 12, TgfbI – Transforming Growth Factor, Beta-Induced.*

### Loss of Panx1 Function Results in the Accumulation of Pericellular Matrix Around Mouse Myoblasts

ADAMTS versicanases such as ADAMTS5 have been shown to mediate proteolysis of a hyaluronan and versican-rich matrix leading to pericellular matrix clearance enabling migration, cell-cell interaction, and fusion (D’hondt et al., 2010; Stupka et al., 2013). Moreover, a recent report shows that *Adamts5* gene knockdown impairs myoblast fusion *in vitro* (Stupka et al., 2013). Hypothesizing that this gene may play a role in the migration- and fusion-defective phenotypes of Panx1^–/–^ myoblasts, we tested whether impaired Panx1 signaling disrupts pericellular matrix accumulation using an erythrocyte exclusion assay. Consistent with our hypothesis, we observed significantly more pericellular matrix in *Panx1^–/–^* myoblast cultures than in *WT* (Supplementary Figure 3, compare A and B, D). Treatment of *Panx1^–/–^* myoblasts with hyaluronidase blocked erythrocyte exclusion (Supplementary Figures 3C,D) indicating that the pericellular matrix is hyaluronan-based (and thus versican containing). These data suggest that reduced levels of ADAMTS5 and/or other extracellular proteinases in Panx1^–/–^ myoblasts may interfere with fusion by attenuating extracellular protein remodeling.

## Discussion

**FIGURE 7:**
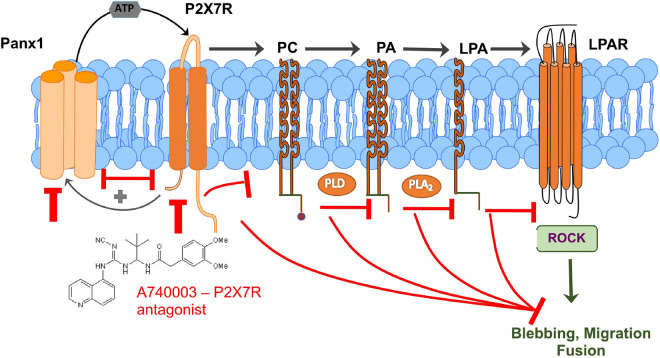
Schematic representation of a new signaling mechanism that connects Panx1/P2X7R signaling with lipid metabolism and lipid-mediated ROCK signaling and regulation of myoblast blebbing/migration/fusion which is important for efficient muscle regeneration. Panx1-regulated P2X7 receptors can signal through PLD and A_2_ (PLA_2_); the resulting lysophosphatidic acid (LPA) then causes dynamic membrane blebbing via a pathway dependent on Rho-associated kinase (ROCK). Interaction between Panx1 and P2X7 receptors leads to further activation of the pannexin channel through a positive feedback loop that has been reported previously (Pelegrin and Surprenant, 2006; Velasquez and Eugenin, 2014). Perturbation of any component of these signaling pathways results in decreased blebbing and reduced myoblast migration and fusion.

In this study, we used genetic perturbation to show for the first time that Panx1 is important for efficient skeletal muscle regeneration *in vivo*. Moreover, *in vitro* studies provided evidence that Panx1 regulates three major satellite cell/myoblast functions: migration, fusion, and remodeling of the ECM, all of which can contribute to formation of myotubes/myofibers. The signaling events downstream of Panx1 that could control these cellular processes were probed using chemical tools. These data supported a model in which Panx1 functions to induce membrane blebbing and migration via activation of the purinergic receptor P2X7, which in turn leads to production of extracellular ATP. Moreover, several lines of evidence support a role for lipid intermediates in the signaling pathway. In particular, myoblast blebbing and migration could be blocked by inhibition of LPA production, and conversely, P2X7 blockade could be rescued by addition of LPA. Based on this data, we propose that Panx1-regulated P2X7 receptors may signal through PLD and A_2_ (PLA_2_); the resulting LPA then causes dynamic membrane blebbing. This pathway could involve Rho-associated kinase (ROCK; Jia et al., 2006; Figure 7). This would be consistent with previous studies showing that ROCK promotes rearrangement of the actin cytoskeleton to facilitate migration and fusion (Signorello and Leoncini, 2014; Hyder et al., 2015).

Blebbing involves changes in the membrane and the underlying actin cytoskeleton. As reported previously, blebs on the surface of myoblasts and other cells are spherical membrane protrusions that appear due to hydrostatic pressure in the cytoplasm but their retraction involves actomyosin complexes of the cell cortex (Charras and Paluch, 2008; Tinevez et al., 2009; Otto et al., 2011). The profound effect of *Panx1* gene ablation or P2X channel blockade on the shape and length of blebs, suggests that Panx1 influences the actomyosin bleb cortex. In addition to the potential for ROCK mediated effects on actin, Panx1/P2X7R may function via direct physical links to the cytoskeleton (Wicki-Stordeur and Swayne, 2013; Boyce et al., 2014). In particular, this complex was shown to directly interact with actin filaments (Kuehnel et al., 2009a,b; Wicki-Stordeur and Swayne, 2013; Boyce et al., 2014), and in BICR-M1R_*k*_ cells, Panx1 has been reported to co-localize and interact with actin at the leading edge of the lamellipodia and filopodia (Jaenisch and Bird, 2003). It was also shown that in the brain PANX1 controls actin cytoskeleton rearrangement during cell migration via interaction with the ARP2/3 complex (Wicki-Stordeur and Swayne, 2013; Boyce et al., 2014). The question of whether Panx1 interacts with directly or indirectly with actin in myoblasts and whether it helps control bleb retraction would be interesting to study in the future.

In addition to cytoskeletal changes that could modulate migration and fusion, the blebbing process might play a direct role in promoting interaction between the plasma membranes of adjacent cells. This idea is supported by recent studies, which demonstrate that blebbing promotes fusion of cancer cells exposed to chemical stress. In this context, bleb-mediated fusion results in the formation of blebbishields that ensure cell survival and growth (Kuehnel et al., 2009b; Jinesh et al., 2013, 2016; Jinesh and Kamat, 2016). We suggest that bleb-mediated myoblast fusion could involve a process that is normally found in cells undergoing apoptosis: plasma membrane scrambling (Marino and Kroemer, 2013). This scrambling involves the formation of dynamic membrane blebs with phosphatidylserine translocated from the inner to the outer membrane. In contrast to the apoptotic pathway, we suggest that in myoblasts these molecules could become available to bind the phosphatidylserine receptor BAI1 on adjacent myoblasts to activate the ELMO/DOCK180/Rac1 pathway leading to fusion (Hochreiter-Hufford et al., 2013).

The precise role of ATP production in Panx1/P2X channel mediated signaling in myoblasts is yet to be defined. ATP is known to mediate paracrine cell-cell signaling (Iglesias and Spray, 2012) and is involved in multiple important biological functions, such as sympathetic nerve activation in the ischemic heart (Dong et al., 2016), leukocyte emigration through blood endothelium (Lohman et al., 2015), and response to mechanical pressure (Lopez et al., 2021). Our new data suggests that ATP release can also promote cell fusion, however, further studies are required to confirm this hypothesis.

While this discussion has focused in the role of Panx1 signaling in blebbing and actin/membrane changes that may promote migration and fusion, it is important to note that myoblast differentiation involves multiple steps including cell cycle exit and commitment to differentiation, myoblast migration and fusion, and sequential expression of differentiation factors (Zammit et al., 2006). A previous study (Langlois et al., 2014), reported that Panx1 is involved in muscle differentiation, in part because they found that the formation of MyHC-expressing myotubes was perturbed by CBX application. However, the study did not define the specific stage of myoblast differentiation controlled by Panx1. Our data indicate that Panx1 inhibition impairs myoblast migration and fusion, but does not alter the expression of differentiation markers. Further work will be required to clarify whether Panx1 has a role in regulation of the muscle regulatory factor (MRF)-driven gene expression program during differentiation.

While levels of core differentiation factors did not seem to be altered in our genetic and chemical Panx1 perturbation studies, we did find that *Panx1^–/–^* myoblasts had reduced expression of matrix remodeling genes including *Adamts5* and MMPs. There was also dysregulation of several adhesion related molecules. A publication from Stupka and co-authors showed the importance of Adamts versicanases and especially Adamts5 in myoblast fusion. Moreover, our data suggested that Panx1 inhibition leads to the failure to remodel pericellular matrix during fusion. Adhesive interactions also play critical roles in migration, proliferation, and fusion of myoblasts, and aberrations in such interactions can lead to compromised function and pathology (Siegel et al., 2009; Lukjanenko et al., 2016). Integrin β (*Itgb1*) expression was reduced in *Panx1^–/–^* myoblasts; *Igtb1*-deficient myoblasts (see Table 1) were previously shown to have defective plasma membrane breakdown and fusion (Schwander et al., 2003). It is possible that reduced expression of several adhesion and matrix remodeling factors (such as *Itgb1* and *Adamts5*) jointly contribute to delayed fusion of *Panx1^–/–^* myoblasts.

In conclusion, our study provides multiple lines of evidence that Panx1 mediates purinergic signaling, which can be mediated via lipid intermediates, to control key processes underlying myoblast migration and fusion including membrane blebbing, ECM remodeling, migration, and adhesion. These pathways are essential for robust skeletal muscle regeneration.

## Data Availability Statement

The original contributions presented in the study are included in the article/Supplementary Material; further inquiries can be directed to the corresponding author/s.

## Ethics Statement

The animal study was reviewed and approved by Scripps Research Institute Animal Care and Use Committee.

## Author Contributions

KS-B, HC-H, AG, and TEW: collection and/or assembly of data, data analysis and interpretation. RM: collection and/or assembly of data, data analysis and interpretation, manuscript writing, and final approval of manuscript. AS: collection and/or assembly of data, data analysis and interpretation, and final approval of manuscript. PRD and SV: data analysis and interpretation. VIS: data analysis and interpretation, conception and design. KP: conception and design, collection and/or assembly of data, data analysis and interpretation, manuscript writing, and final approval of manuscript. HPM: conception and design, collection and/or assembly of data, data analysis and interpretation, manuscript writing, final approval of manuscript, financial support, administrative support, and final approval of manuscript. All authors contributed to the article and approved the submitted version.

## Conflict of Interest

The authors declare that the research was conducted in the absence of any commercial or financial relationships that could be construed as a potential conflict of interest.

## Publisher’s Note

All claims expressed in this article are solely those of the authors and do not necessarily represent those of their affiliated organizations, or those of the publisher, the editors and the reviewers. Any product that may be evaluated in this article, or claim that may be made by its manufacturer, is not guaranteed or endorsed by the publisher.

## Supplementary Material

Supplementary Figure 1 *Panx1^–/–^* myoblasts obtained from *Panx1KO/B6* mice show significant delays in myotube formation, confirming the phenotype of *Panx1^–/–^* myoblasts from *CMV-Cre/Panx1^*flf/fl*^* mice; *Casp11^–/–^* myoblasts show normal differentiation. **(A)** *Casp11^–/–^* myoblasts: *Casp11^–/–^* myoblasts have prominent surface blebs and differentiate similarly to WT myoblasts at 24 and 48 h in DM. **(B)** *Panx1^–/–^* myoblasts: *Panx1^–/–^* myoblasts have fewer surface blebs and do not spread well through the surface of the dish. *Panx1^–/–^* myoblasts show a delay in myotube formation at 24 h **(B,C)** and 48 h after replacement of GM with the DM. **(E–H,I)** Fusion index quantification of *Panx1^–/–^* (*Panx1KO/B6*), *WT*, and *Casp11^–/–^* myoblasts. Myoblast fusion was calculated as the percentage of MyHC expressing cells containing one, two, or ≥3 nuclei at 48 h after induction of differentiation. Data are presented as mean ± standard deviation. *n* = 3 independent experiments. ^∗^*P* < 0.05 compared with WT.

Supplementary Figure 2 Western blot analysis **(A)** and quantification **(B)** of the MyCH in control (WT untreated) myoblasts and myoblasts treated with the 10 mm and 25 mm of CBX. Analysis of MyCH expression was perfomed 1 and 48 h after induction of differentiation. Control is vehicle treated myoblasts. Myoblast Lysate was probed with anti-MyCH antibody at 1:1000 dilution (#05-716, clone A4.1025, Sigma-Aldrich).

Supplementary Figure 3 Loss of Panx1 function in primary myoblasts results in accumulation of pericellular matrix **(A,B)**, *Panx1^–/–^* myoblasts (**A**-red dashed line) accumulate more pericellular matrix as shown by the exclusion of erythrocytes (yellow dashed line) than WT myoblasts **(B)**. Treatment of *Panx1^–/–^* myoblasts with hyaluronidase (hyalur) reduces the area of erythrocyte exclusion to be similar to WT myoblasts **(C)**. *Scale bar* = 15 μm. **(D)** Quantitation of the area of pericellular matrix exclusion around WT myoblasts and *Panx1^–/–^* myoblasts with and without hyaluronidase. Data is the ratio of exclusion area to cellular area; images are taken randomly from three independent experiments (*n* ∼20 cells per condition). ^∗^*p* < 0.001.
