# Supplementary figures and images for: Pannexin 1 Regulates Skeletal Muscle Regeneration by Promoting Bleb-Based Myoblast Migration and Fusion Through a Novel Lipid Based Signaling Mechanism
Source: Front Cell Dev Biol. 2021 Oct 5;9:736813. doi: 10.3389/fcell.2021.736813 (PMC8523994; doi:10.3389/fcell.2021.736813)

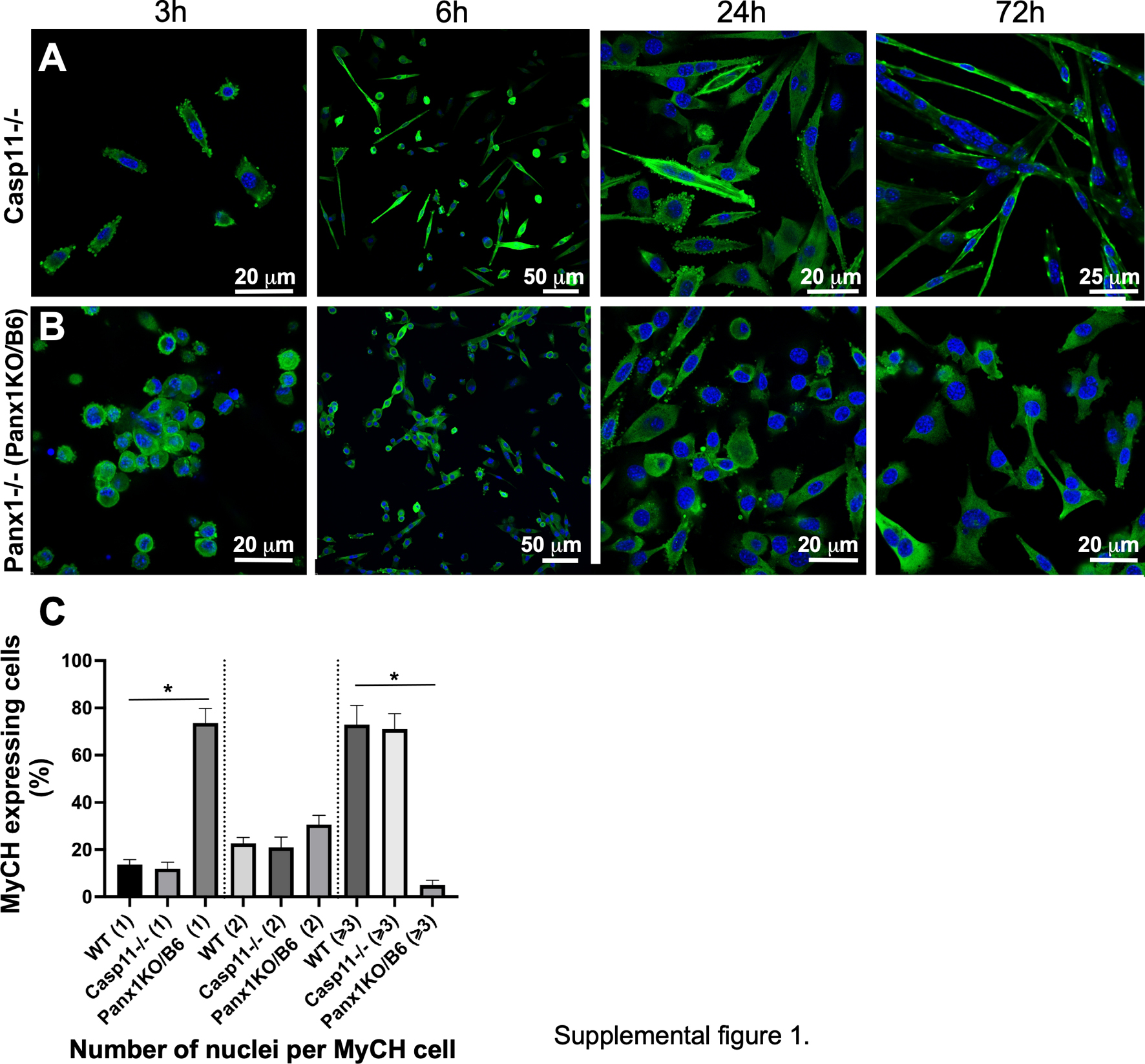

Supplement: Supplementary Figure 1 — Panx1–/– myoblasts obtained from Panx1KO/B6 mice show significant delays in myotube formation, confirming the phenotype of Panx1–/– myoblasts from CMV-Cre/Panx1flf/fl mice; Casp11–/– myoblasts show normal differentiation. (A) Casp11–/– myoblasts: Casp11–/– myoblasts have prominent surface blebs and differentiate similarly to WT myoblasts at 24 and 48 h in DM. (B) Panx1–/– myoblasts: Panx1–/– myoblasts have fewer surface blebs and do not spread well through the surface of the dish. Panx1–/– myoblasts show a delay in myotube formation at 24 h (B,C) and 48 h after replacement of GM with the DM. (E–H,I) Fusion index quantification of Panx1–/– (Panx1KO/B6), WT, and Casp11–/– myoblasts. Myoblast fusion was calculated as the percentage of MyHC expressing cells containing one, two, or ≥3 nuclei at 48 h after induction of differentiation. Data are presented as mean ± standard deviation. n = 3 independent experiments. ∗P < 0.05 compared with WT. [file Image_1.JPEG]

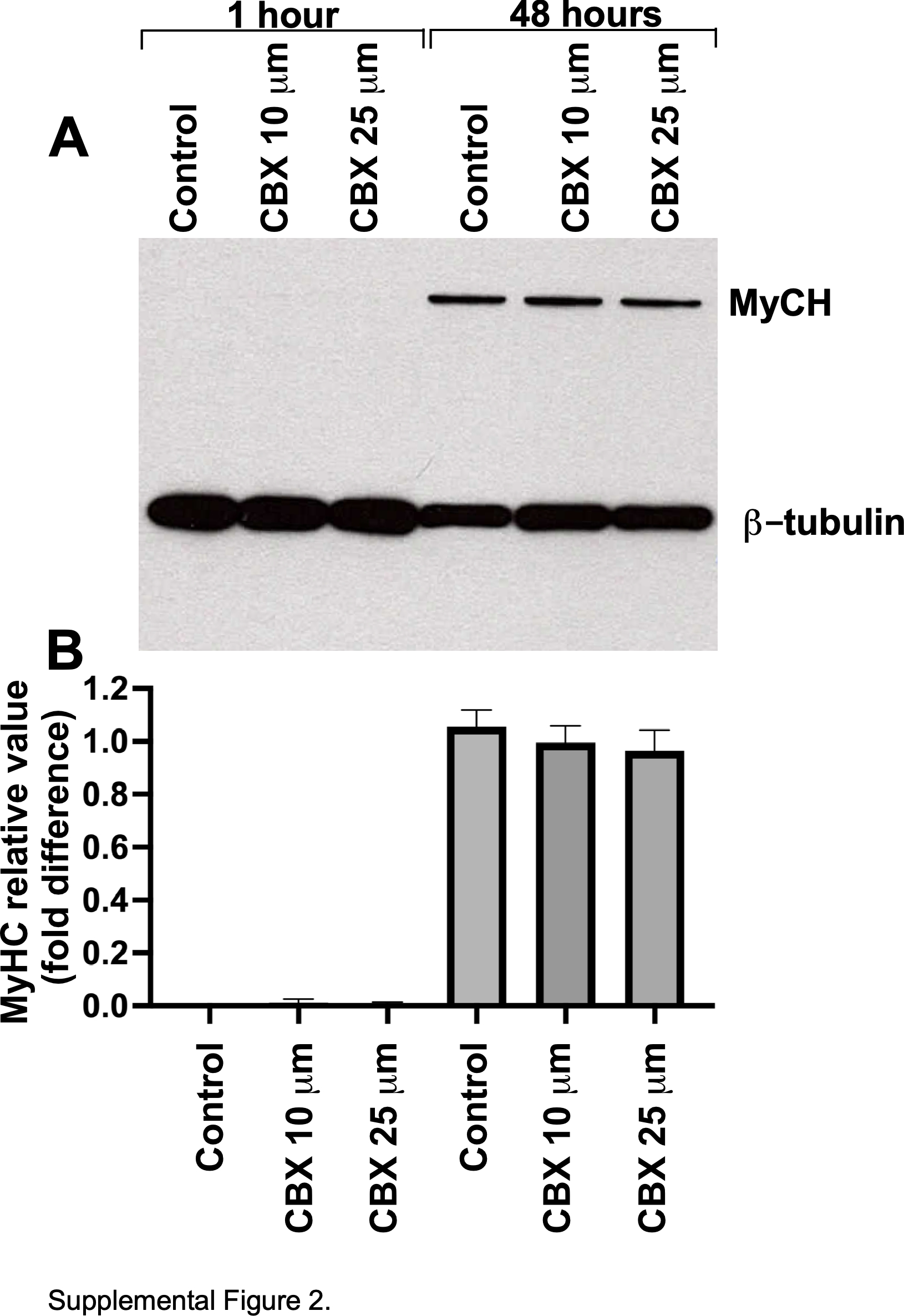

Supplement: Supplementary Figure 2 — Western blot analysis (A) and quantification (B) of the MyCH in control (WT untreated) myoblasts and myoblasts treated with the 10 mm and 25 mm of CBX. Analysis of MyCH expression was perfomed 1 and 48 h after induction of differentiation. Control is vehicle treated myoblasts. Myoblast Lysate was probed with anti-MyCH antibody at 1:1000 dilution (#05-716, clone A4.1025, Sigma-Aldrich). [file Image_2.JPEG]

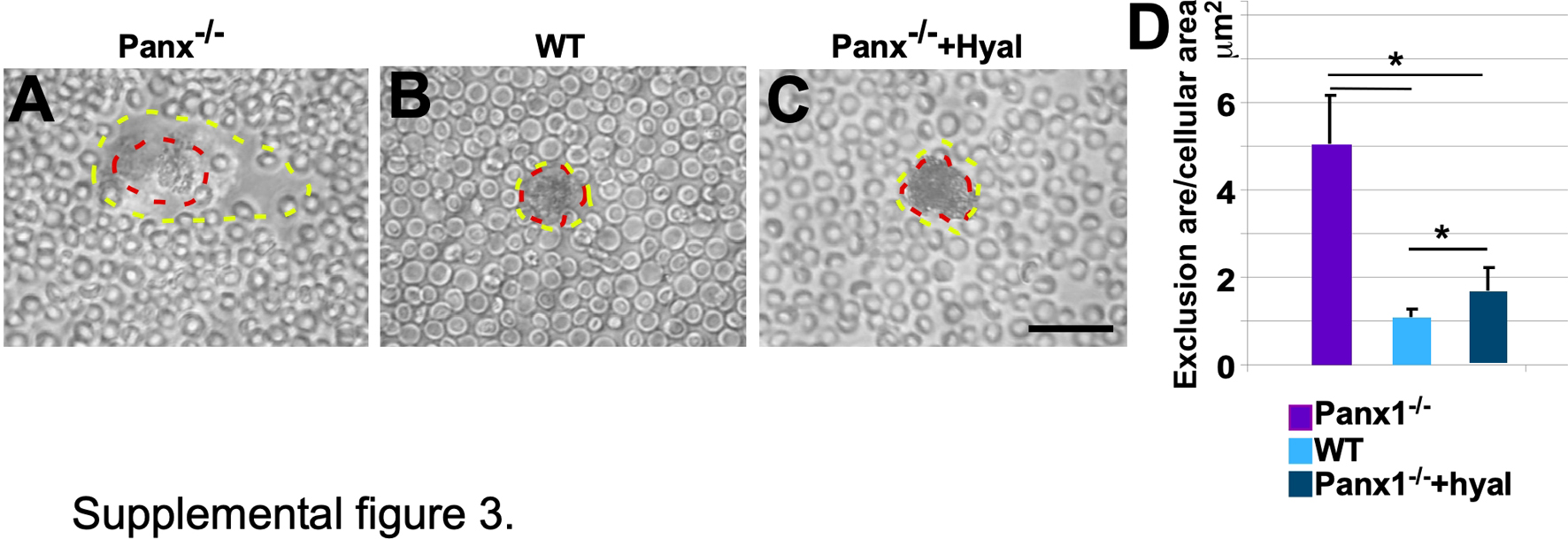

Supplement: Supplementary Figure 3 — Loss of Panx1 function in primary myoblasts results in accumulation of pericellular matrix (A,B), Panx1–/– myoblasts (A-red dashed line) accumulate more pericellular matrix as shown by the exclusion of erythrocytes (yellow dashed line) than WT myoblasts (B). Treatment of Panx1–/– myoblasts with hyaluronidase (hyalur) reduces the area of erythrocyte exclusion to be similar to WT myoblasts (C). Scale bar = 15 μm. (D) Quantitation of the area of pericellular matrix exclusion around WT myoblasts and Panx1–/– myoblasts with and without hyaluronidase. Data is the ratio of exclusion area to cellular area; images are taken randomly from three independent experiments (n ∼20 cells per condition). ∗p < 0.001. [file Image_3.JPEG]
